# Microelectrode Arrays and the Use of PEG-Functionalized Diblock Copolymer Coatings

**DOI:** 10.3390/bios4030318

**Published:** 2014-09-11

**Authors:** Sakshi Uppal, Matthew D. Graaf, Kevin D. Moeller

**Affiliations:** Department of Chemistry, Washington University in St. Louis, St. Louis, MO 63130, USA; E-Mails: suppal@wustl.edu (S.U.); mgraaf@wustl.edu (M.D.G.)

**Keywords:** microelectrode arrays, PEG, ferrocene carboxylic acid, cyclic voltammetry

## Abstract

PEG-modified diblock copolymer surfaces have been examined for their compatibility with microelectrode array based analytical methods. The use of PEG-modified polymer surfaces on the arrays was initially problematic because the redox couples used in the experiments were adsorbed by the polymer. This led the current measured by cyclic voltammetry for the redox couple to be unstable and increase with time. However, two key findings allow the experiments to be successful. First, after multiple cyclic voltammograms the current associated with the redox couple does stabilize so that a good baseline current can be established. Second, the rate at which the current stabilizes is consistent every time a particular coated array is used. Hence, multiple analytical experiments can be conducted on an array coated with a PEG-modified diblock copolymer and the data obtained is comparable as long as the data for each experiment is collected at a consistent time point.

## 1. Introduction

Microelectrode arrays have great potential for monitoring interactions between the members of a molecular library and biological receptors because every electrode in the array is individually addressable [1,2,3,4,5,6,7,8,9,10,11,12,13,14]. Hence, if the molecules in a library are placed (or synthesized) on a microelectrode array such that each unique member of the library is located next to a unique, addressable set of electrodes, then each member of the array can be independently monitored. The process requires three key steps. First, the array is coated with a stable, porous polymer that provides the functionality needed to attach molecules to the surface of the electrodes in the array [15]. Second, synthetic methods that operate selectively at individual electrodes in the array are used to either add or build molecules on the array proximal to the electrodes that will be employed to monitor their behavior [16,17]. Third, the electrodes in the array are used to establish and then monitor the current associated with a redox couple placed in the solution above the array. Once this is accomplished, treatment of the array with a receptor causes a change in the current associated with the redox mediator at the electrodes next to the molecules in the library that bind [18,19]. This change in current is recorded. The result is a method that allows for the molecules on the array to be monitored in “real-time” without labeling either the molecule or the receptor being studied [18,19,20,21,22,23,24,25,26,27,28,29,30,31,32,33,34].

**Figure 1:**
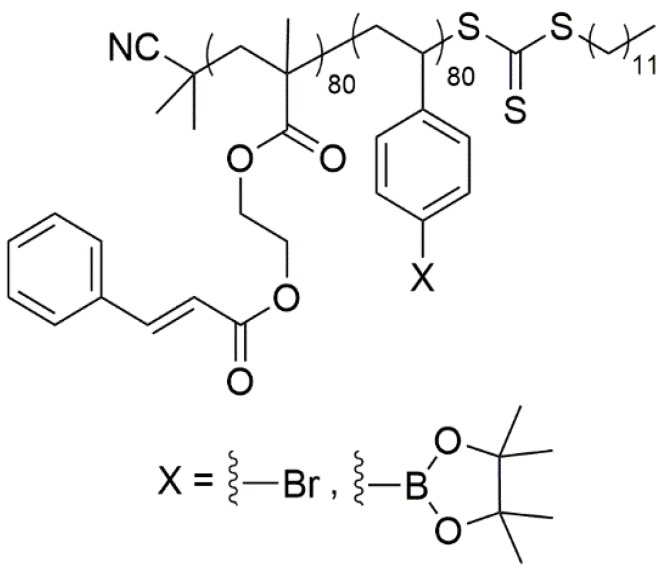
Diblock copolymer for coating arrays.

Central to this effort is the polymer used to coat the array [15]. Two main types of experiments define the nature of this polymer. First, for the analysis of a larger library it is best to synthesize the molecules on the arrays instead of transfer them to the array one at a time. Hence, the surface of the array must not only provide a platform for the attachment of an initial substrate, but also it must be inert to the range of chemistry employed in subsequent steps. Second, the arrays are being developed so that biological data can be gathered quickly and then used to guide the synthesis of molecules in smaller, targeted libraries. In these experiments, molecules are synthesized, added to a growing library on an array, and then analyzed for activity relative to the other members of the library. For this strategy, the surface of the array must be stable so that the library on the array can be reused multiple times over an extended period of time. To meet these needs, we have found that diblock copolymers having the general structure of **1** (Figure 1) to be very effective. The polymers are comprised of one block of a cinnamate functionalized methacrylate that is crosslinkable and used to impart stability to the surface, and one block of a functionalized polystyrene block that is used as attachment points for fixing molecules to the surface of the electrodes in the array. The resulting surfaces are stable and allow for the use of nucleophiles, electrophiles, oxidants, reductants, acids, bases, and transition metal catalysts on the arrays. 

While the use of the diblock copolymer is ideal in many ways, it does force the use of an indirect method for the detection of binding-events that occur on the surface of the array. Molecules bound to the surface of the polymer cannot be observed directly. Thus, as described above, binding events on the array are monitored with the use of a solution phase redox couple. The need for this redox couple (typically iron) [18,19] led to concerns about efforts to pacify the surface of the array toward nonspecific binding with proteins, and how those efforts might alter subsequent analytical experiments. Will groups added to the surface of the array in order to reduce nonspecific binding events bind the iron redox couple used for the analytical studies and in so doing interfere with the analysis? This fear was quickly realized when the surface of the diblock copolymer was modified with the poly(ethylene glycol) (PEG) polymers frequently used to minimize nonspecific binding events between surfaces and proteins [35,36,37]. The PEG-modified diblock copolymer bound the iron redox couple used in the signaling experiments and led to CV data for the iron that steadily increased in current over time. A baseline CV for conducting a binding study could not be obtained, and the effort was initially abandoned. Fortunately, later studies showed that drifts in current of this nature often stabilize with sufficient time [15]. We report here that this is true for an array coated with a PEG-modified diblock copolymer, and that the drift in current for this system increases at a consistent rate. Based on these findings, a microelectrode array coated with a PEG-functionalized diblock copolymer can be used for a series of analytical studies if the current data for each experiment is collected at a consistent point in time. 

## 2. Experimental Section 

Materials were purchased from Sigma Aldrich unless specified otherwise and were used without further purification. The diblock copolymer polycinnamoyloxy ethyl methacrylate-b-poly-4-pinacolatoborylstyrene (PCEMA-b-pBSt/Figure 1, X = Bpin) was prepared as previously reported [15].

### 2.1. Procedure for Coating Arrays with Block Copolymer

The microelectrode arrays (containing 12,544 electrodes/cm^2^) were spin-coated using a MODEL WS-400B-6NPP/LITE spin-coater available from Laurell Technologies Incorporated. In this effort, three drops of 0.03 g/mL block copolymer solution PCEMA-b-pBSt in 4:1.5 DMF/THF) were placed on the array making sure that the entire electrode area of the array was covered. The array was then spun at 1000 rpm for 40 s. The block copolymer coating was allowed to dry for 20 min and then cross-linked using a 100W Hg lamp for 20 min before use.

### 2.2. Procedure for Conducting Cyclic Voltammetry Experiments on a 12-K Array

Cyclic voltammetry was carried out on a BAS 100B Electrochemical Analyzer potentiostat, with BAS 100W version 2.31 control software. A 12-K microelectrode array was cleaned with Nano-strip (Cyantek Corporation) and spin coated with the block copolymer solution as mentioned above. The polymer can then be functionalized next to the electrodes as described previously [16]. To the array is then added 120 µL of an iron solution (either 5 mM ferrocene carboxylic acid or 8 mM ferrocyanide and 8 mM ferricyanide) in 20 mM HEPES (4-(2-hydroxyethyl)-1-piperazineethanesulfonic acid, pH 8.0) with 0.1 M potassium nitrate that contained a predetermined concentration of Bovine Serum Albumin (BSA) or any protein of interest. The array was then placed in an ElectraSense reader [2] and one block of 12 electrodes was activated. Cyclic voltammetry measurements were made by sweeping the potential at selected electrodes on an array from −800 to 800 mV at a scan rate of 400 mV/s. The potential in these experiments refers to the difference in potential of the working electrode relative to the counter electrode, a platinum plate of area 0.75 cm^2^ located approximately 650–800 µm away from the array and held in place by an O-ring. Each experiment was conducted at three independent blocks of 12 electrodes each chosen randomly from different areas of the array. After the measurement was made for a concentration of BSA in solution, the array was washed with the next concentration of BSA by injecting and withdrawing the solution three times. Detailed protocols on operating the microelectrode arrays have been published [17].

As an alternative, the data in the study above could be collected after either one or ten CV scans without allowing the current measurement to fully equilibrate. Since the initial change in current occurred at a consistent rate, the same overall binding data for BSA to the surface of the array could be obtained at any time point as long as the same time point was used each time. 

### 2.3. PEGylation of PCEMA-b-pBSt Polymer Using Chan-Lam Coupling on Arrays

The following procedure was adapted from the known Chan-Lam coupling [15,38]. Poly(ethylene glycol) methyl ether-750 (10.0 mg) and 50.0 mg of tetrabutylammonium hexafluorophosphate were dissolved in 1.0 mL of DMF. To this solution, 50.0 µL of a 25 mM copper(II) acetate in water solution was added. Contents were mixed, and then 120 µL of this solution was added to the array. The array was placed into an ElectraSense reader and all 12,544 electrodes selected and used as anodes. A potential of +2.4 V relative to the auxiliary electrode was used to pulse the electrodes for 20 cycles of 30 s on and 10 s off. After completion of reaction, the array was washed extensively with 95% ethanol. 

**Scheme 1:**
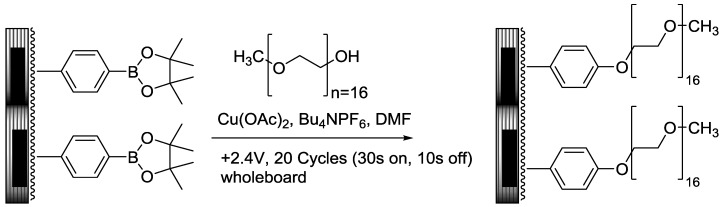
Chan-Lam type coupling reaction on a microelectrode array.

## 3. Results and Discussion

Most of our early work with block copolymers utilized a bromine substituted polystyrene block (Figure 1, X = Br) to attach molecules to the array. It was this polymer that was functionalized with PEG in the original studies described in the introduction. With time and a desire to build tunable surfaces on the arrays [15], the block copolymer was changed to one that contained a borate ester substituted polystyrene block (Figure 1, X = Bpin). While synthetically advantageous, the use of the borate ester on the array initially led to the same issues we had seen with the PEG functionalized surface. The current measured for a ferricyanide redox mediator on an array coated with the borate ester polymer was not stable [15]. In playing with the borate ester surface, it was eventually discovered that after repetitive CV scans the current associated with the redox mediator would stabilize. After that point, the peak current for the CV could be measured and used to monitor binding events that occurred over the electrodes in the array. We reasoned that if the same scenario occurred with PEG functionalized surfaces, then PEG functionalized block copolymers might also be compatible with microelectrode array-based signaling studies. 

To test this idea, a microelectrode array was spin coated with PCEMA-b-pBSt (X = Bpin), photolyzed to crosslink the cinnamate esters and add stability to the surface, and then functionalized with a PEG polymer having 16 repeating units (n = 16) and a methyl ether on one end (PEG-16). The chemistry took advantage of the Chan-Lam coupling method previously developed to place alcohols on the surface of the array above the electrodes. In this reaction, a Cu(II)-PEG complex undergoes an electrophilic substitution on the aromatic ring that exchanges the borate ester for Cu(II)-PEG. A reductive elimination then couples the PEG to the aryl ring. The electrode is then used to regenerate the Cu(II)-catalyst. 

The array was then used for signaling experiments with both ferrocene carboxylic acid (FCA) and ferricyanide/ferrocyanide as the redox couple. While the ferricyanide/ferrrocyanide couple has been used most frequently in the studies reported to date [18,19], with the PEG-modified polymer surface on the array the use of the FCA redox couple consistently led to lower variance (smaller error bars) in the binding data generated. Hence, all of the experiments discussed below were conducted with FCA as the redox mediator. 

**Figure 2:**
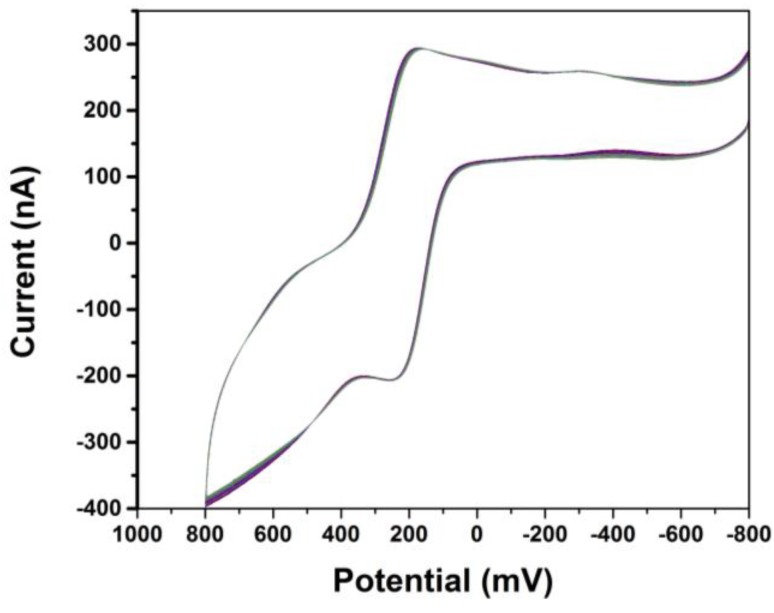
CV for FCA on a microelectrode array (scans 20–40).

The first experiment attempted examined whether or not the currents measured on the PEG functionalized surface would stabilize in the same manner that the currents did on non-PEG functionalized borate ester surface. When a microelectrode array was functionalized by every electrode with PEG-16 and then covered with a buffer solution containing FCA and potassium nitrate electrolyte, the current measured for the FCA during CV experiments initially rose as expected with each run. Just as in the case of the non-PEG functionalized borate ester polymer, the current measured eventually stabilized after collecting about 20 voltammograms. The stability of the wave following this period was shown by running a total of 65 CV’s with no further change. In Figure 2, CV scans 20–40 of this effort are shown in order to illustrate that the stabilized wave was very reproducible. A baseline for subsequent signaling studies had been established.

**Figure 3:**
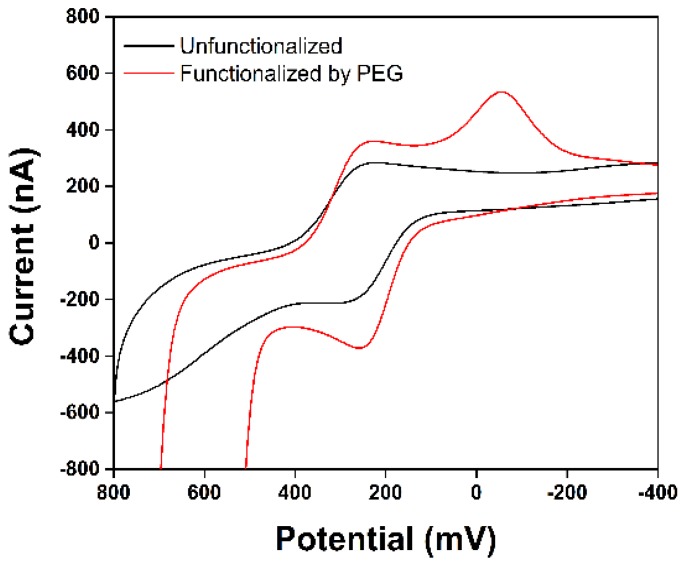
Red line—CV for FCA with the PEG functionalized polymer coating. Black line —CV for FCA with the unfunctionalized polymer.

Functionalization of the diblock copolymer with PEG-16 led to an increase in the magnitude of the current measured for FCA and a change in the overall shape of the wave relative to that obtained with the non-PEG functionalized surface (Figure 3). Both observations can be explained by the change in the nature of the polymer caused by incorporation of the hydrophilic PEG-16 group. The more hydrophilic polymer swells better in water, a situation that allows higher adsorption of the iron species into the polymer leading to a higher peak current. This increase in peak current is beneficial for the subsequent signaling experiments because it makes it easier to detect changes in the current when binding events occur on the surface of the electrode. 

**Figure 4:**
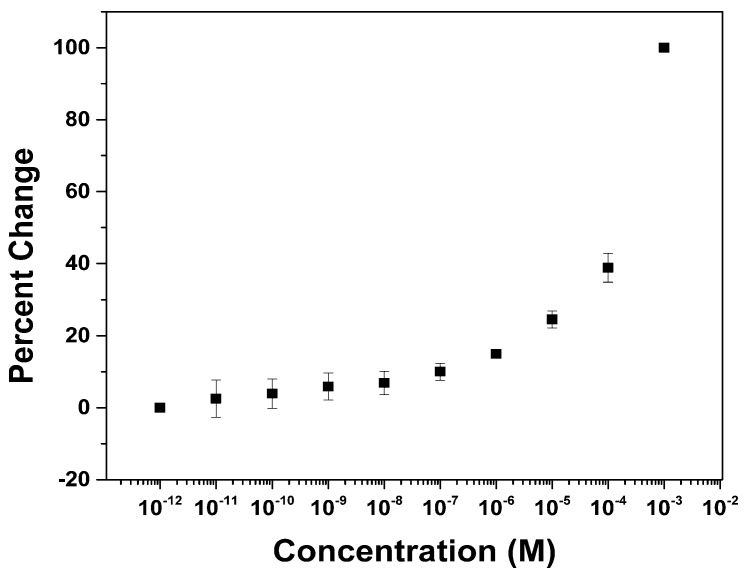
Curve for the nonspecific binding of BSA to an array coated with a PEG functionalized polymer.

The compatibility of the PEG-16 modified surface with array-based signaling studies was examined by looking at the nonspecific binding of bovine serum albumin (BSA) to the polymer coating on the array. To this end, an array coated with the PEG-16 modified diblock copolymer was placed in a 1 picomolar solution of BSA in HEPES buffer that contained FCA and potassium nitrate as an electrolyte. A block of twelve electrodes on the array was used to collect cyclic voltammetry data for the FCA. In the first trial, thirty cyclic voltammograms were run in order to make sure that the current stabilized. The anodic current for the oxidation of Fe^2+^ was then measured by noting the difference between the current at the peak of the CV wave and the baseline current leading into the wave. This procedure was repeated using two additional blocks of twelve electrodes. The current obtained from each of the three blocks of electrodes was then averaged and recorded. Following the experiment with 1 picomolar BSA, the array was washed three times with an electrolyte solution that contained 10 picomolar BSA. The CV experiment was then repeated in triplicate using the new concentration of BSA. Again, the peak current was measured after 30 CV scans. This procedure was then repeated each time increasing the concentration of the receptor in solution by a factor of ten. The experiment was stopped when a concentration of one millimolar BSA was reached. The data obtained was then plotted on a chart that compared the relative current change to the concentration of BSA in solution (Figure 4). The data in the chart was normalized by assigning the highest current measured on the array a value of zero and the lowest current on the array a value of one. In this way, the larger the percent change the larger the binding of BSA to the surface. The error bars shown in Figure 4 show the spread in the data recorded at the three separate sites on the array for each concentration of BSA.

The nonspecific binding profile for BSA to the PEG modified polymer surface can be seen in Figure 4. Clearly, the use of the PEG-16 functionalized polymer is compatible with the experiment. The success of the approach led to two immediate questions. First, can a binding profile for the nonspecific binding event be obtained before the CV wave stabilizes, and second, is the experiment compatible with the longer PEG-polymers?

**Figure 5:**
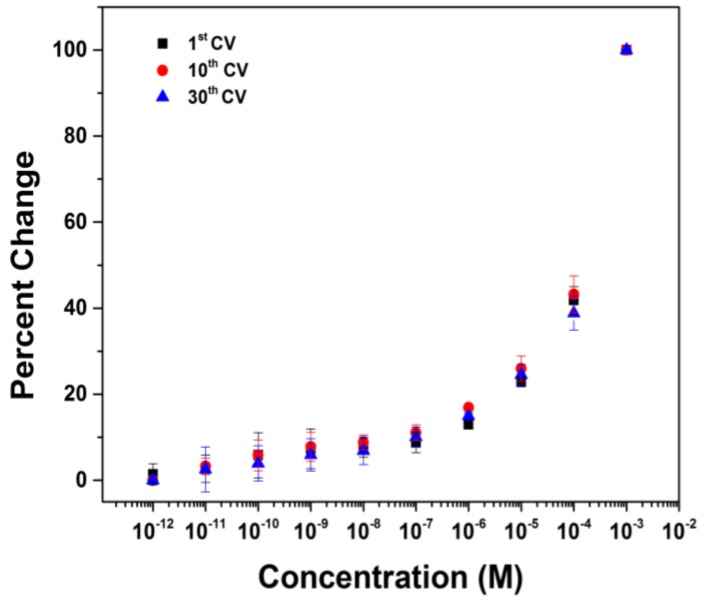
Binding curves generated at different time points.

The first question was important because protein stability can be an issue. In such cases, it would be better if the analytical experiment took less time. With this in mind, we wondered if the rate at which the current in the CV experiments stabilized was constant. If so, then the current data for an analytical experiment could be obtained by taking the data for each concentration of protein used at the same point in time. This turned out to be the case. In Figure 5, the nonspecific binding of BSA to the surface is shown for three different experiments. In one (a repeat of Figure 4), the peak current used to generate the graph was taken from the 30th CV scan at each concentration of BSA. Again, this was to make sure that the system would be fully equilibrated and a stable current obtained. In the second, the peak current for the 10th CV scan at each concentration of BSA was used to generate the binding curve. In the third, the current for the first CV scan at each concentration of BSA was used. The goal of this experiment was to determine if the drift in current could be completely ignored. While the raw CV data in the three experiments did vary significantly, the change in that data relative to the amount of BSA present did not. All three experiments generated the same binding curve for BSA to the surface. 

**Figure 6:**
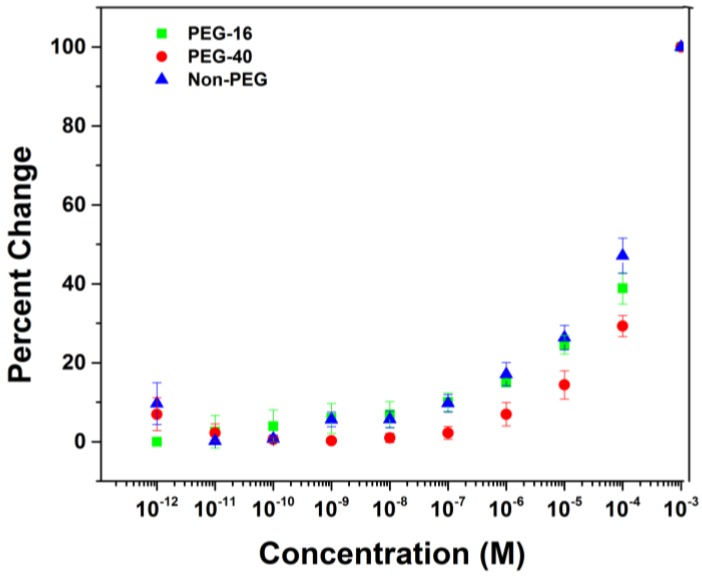
Comparison of polymers functionalized with different lengths of PEG.

The second question about the use of longer PEG-polymer was asked because longer PEG-polymers are often required to pacify the nonspecific binding of a protein to a surface [37]. To test the idea, an array coated with the diblock copolymer was functionalized with a PEG mono methyl ether that was made of 40 repeat units (PEG-40). The experiment to measure nonspecific binding events on the surface was repeated using the exact same protocol used for the PEG-16 modified surface. The data was plotted along with the data obtained with the PEG-16 surface and the data obtained with an unfunctionalized array (Figure 6). The signaling experiment with the array coated with the PEG-40 modified polymer worked very well. The curve for the nonspecific binding of BSA to the surface (red dots) was nearly identical to the curve obtained with either the PEG-16 modified polymer or the unmodified system. The only difference was that the surface functionalized with the PEG-40 modified polymer showed lower nonspecific binding than did the other alternatives. Note how at each concentration of BSA greater than 10^−7^ M the relative change for the PEG-40 modified problem was significantly smaller than the other two curves. In this way, the use of the longer PEG was not only compatible with the experiment, but also more successful in pacifying the surface to nonspecific interactions with BSA.

At this time, the exact origin of the difference between the PEG-16 and the PEG-40 is not known. We do not have an accurate assessment of surface coverage. Placing PEG on the surface of the array swells the polymer and makes available more potential binding sites for small fluorescent probes that might be used to make that assessment. Hence, it is possible that the actual surface coverage is low. In that case, it might be that the greater pacification of the surface with PEG-40 is simply due to its larger size. Efforts to understand this and optimize the coverage of PEG on the surface are underway. 

## 4. Conclusions

PEG-modified diblock copolymer coated microelectrode arrays can be used in analytical experiments that monitor the current associated with an iron redox couple as long as the experiments are conducted at a consistent point in time. That point in time can be the first CV scan taken, a scenario that minimizes the overall time required for conducting an analysis. These findings open the door for the use of PEG-polymers to pacify nonspecific binding events on microelectrode arrays coated with porous block copolymer reaction layers. 
